# Acupuncture for Poststroke Dysphagia: An NTS‐Centered Hypothesis‐Generating Framework Linking the Brainstem Swallowing Network, Neuroimmune Regulation, and Vagal Anti‐Inflammatory Signaling

**DOI:** 10.1002/brb3.71732

**Published:** 2026-09-17

**Authors:** Wei Li, Lebin Liu, Yingying Tu, Chongjiu Gong, Xiao Ran, Jiuguo Pei

**Affiliations:** ^1^ Department of Rehabilitation Medicine Hubei Rongjun Hospital Wuhan China; ^2^ Department of Rehabilitation Medicine The Second Hospital of Huangshi Huangshi China

**Keywords:** acupuncture, neuroimmune regulation, nucleus tractus solitarius, poststroke dysphagia

## Abstract

**Background:**

Poststroke dysphagia (PSD) may reflect not only focal motor pathway injury but also instability of the brainstem swallowing network under neuroimmune, glial, and vascular microenvironmental stress.

**Methods:**

This mechanism‐oriented narrative review synthesized evidence on swallowing central pattern generator (CPG) organization, nucleus tractus solitarius (NTS)‐related sensory integration within the medullary swallowing network, poststroke neuroinflammation, altered glial responses, blood–brain barrier/neurovascular unit (BBB/NVU) dysfunction, vagus‐related anti‐inflammatory regulation, and acupuncture‐mediated neuromodulation.

**Results:**

Evidence suggests that inflammatory amplification, altered glial responses, and BBB/NVU instability may be associated with reduced reserve in the distributed medullary swallowing network. Within this broader dorsal and ventral swallowing circuitry, the NTS contributes to sensory integration and premotor organization rather than serving as the sole center of swallowing pattern generation. Such disturbances may contribute to respiratory–swallow discoordination in vulnerable patients. Acupuncture and electroacupuncture may indirectly support swallowing‐network recovery by modifying inflammatory burden, glial state, autonomic balance, and microenvironmental permissiveness. However, most mechanistic evidence is derived from ischemic stroke models in which swallowing was not the primary outcome.

**Conclusion:**

We propose an NTS‐focused, hypothesis‐generating framework in which acupuncture may support PSD recovery by modifying neuroimmune, glial, vascular, and autonomic background conditions relevant to brainstem swallowing‐network stability. This framework should not be interpreted as evidence that acupuncture directly acts through a confirmed NTS–vagal anti‐inflammatory pathway. Future studies should distinguish direct PSD evidence from indirect mechanistic evidence, stratify patients by inflammatory burden and autonomic tone, and incorporate physiological proxies of respiratory–swallow phase stability as mechanistically informative outcomes.

## Graphical abstract

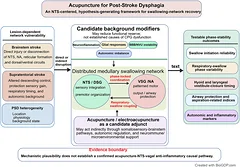

## 1 Introduction

Poststroke dysphagia (PSD) is common after stroke and is linked to aspiration pneumonia, malnutrition, prolonged hospitalization, and mortality (Takizawa et al. 2016). Current care relies on swallowing rehabilitation, compensatory strategies, and prevention of complications (Labeit et al. 2024). Yet recovery remains uneven. Some patients regain safe oral intake early. Others fluctuate or remain unsafe despite standard therapy.

PSD is often framed as a motor execution deficit caused by corticobulbar tract injury or medullary motor‐nucleus involvement (Ertekin and Aydogdu 2003). This view is necessary but incomplete. Dysphagia may follow supratentorial stroke without visible brainstem injury. Symptoms may also fluctuate with respiratory–swallow discoordination and autonomic changes (Labeit et al. 2024). These patterns point to network instability, not only pathway interruption.

Swallowing depends on central pattern generator (CPG)‐mediated integration of sensory input, descending control, respiratory timing, and autonomic background signals (Ertekin and Aydogdu 2003). Within this system, the nucleus tractus solitarius (NTS) integrates glossopharyngeal and vagal afferents and coordinates with the nucleus ambiguus (NA) to support airway protection and pharyngeal propulsion (Ertekin and Aydogdu 2003). This brainstem network has high timing demands and limited redundancy. Small shifts in excitability, glial support, or vascular homeostasis may therefore impair output reliability.

Stroke also induces central and systemic immune responses. These responses can alter remote brain regions through humoral mediators, blood–brain barrier (BBB) dysfunction, and brain–peripheral immune crosstalk (Brea 2023; H. Wang et al. 2023). Even without direct ischemic injury to the NTS or NA, persistent inflammatory burden may reduce CPG reserve and make swallowing more vulnerable to respiratory phase mismatch.

Acupuncture has been reported to influence inflammatory signaling, glial responses, BBB/neurovascular unit (NVU)‐related changes, and autonomic tone in stroke‐related or neurological contexts (Cao et al. 2021; Y.‐W. Li et al. 2022; Ma et al. 2025). However, these findings do not by themselves establish a direct mechanism for swallowing recovery after stroke. In this review, we integrate evidence from swallowing neurophysiology, poststroke neuroimmune disturbance, vagus‐related anti‐inflammatory regulation, and acupuncture‐mediated neuromodulation to develop an NTS‐centered, hypothesis‐generating framework for PSD. We emphasize the distinction between direct swallowing‐related evidence and indirect mechanistic evidence, and propose measurable physiological phenotypes that may help test this framework in future studies.

## 2 Methods

### 2.1 Search Strategy and Study Selection

This review was designed as a mechanism‐oriented narrative synthesis. Relevant literature was identified through targeted searches of PubMed, Web of Science, Embase, and Google Scholar, together with citation tracking from key reviews and original experimental studies. The search was updated in April 2026. Search terms were organized around the following concepts: PSD, swallowing CPG, NTS, NA, brainstem swallowing network, respiratory–swallow coordination, vagus nerve, cholinergic anti‐inflammatory pathway (CAP), neuroinflammation, microglia, astrocytes, BBB, NVU, acupuncture, and electroacupuncture. Representative PubMed search combinations included the following: (“post‐stroke dysphagia” OR “stroke‐related dysphagia”) AND (“nucleus tractus solitarius” OR “nucleus ambiguus” OR “swallowing central pattern generator” OR “brainstem swallowing network”); (“ischemic stroke” OR stroke) AND (neuroinflammation OR microglia OR astrocytes OR “blood–brain barrier” OR “neurovascular unit”); and (acupuncture OR electroacupuncture) AND (“post‐stroke dysphagia” OR swallowing OR stroke) AND (“nucleus tractus solitarius” OR vagus OR autonomic OR neuroinflammation). Equivalent keywords and database‐specific syntax were adapted for Web of Science, Embase, and Google Scholar.

Peer‐reviewed original studies, systematic reviews, and focused narrative reviews were considered eligible when they directly informed at least one predefined domain of the framework. These domains included poststroke swallowing physiology, brainstem swallowing control, NTS‐ and NA‐related circuitry, poststroke neuroimmune and glial responses, BBB/NVU dysfunction, vagus‐related autonomic or anti‐inflammatory regulation, and acupuncture‐ or electroacupuncture‐related neuromodulation. Priority was given to original experimental and clinical studies that directly assessed swallowing outcomes or examined neural, immune, glial, vascular, or autonomic mechanisms relevant to the proposed framework. Reviews were used primarily to define the broader clinical or mechanistic context and to identify additional original studies. Conference abstracts, editorials, non‐peer‐reviewed reports, duplicate publications, and studies without a clear relationship to the predefined domains were not used as core evidence.

The aim of this review was not to estimate treatment efficacy. Instead, it was to integrate evidence from related but not fully overlapping fields and to define a testable mechanistic model for future PSD research. Therefore, no formal meta‐analysis, risk‐of‐bias assessment, or PRISMA‐based selection process was performed. The final synthesis drew on 75 publications. Of these, 21 representative studies are summarized in Table 1 to illustrate the principal evidence domains, degrees of directness, and inference boundaries. Table 1 is intended as a representative evidence map rather than an exhaustive list of the literature identified through the searches.

**TABLE 1:** Evidence categories supporting the NTS‐centered hypothesis‐generating framework for poststroke dysphagia and acupuncture‐related modulation.

Study	Model/subjects	Main focus	Swallowing outcome directly assessed	Evidence type	Relevance and inference boundary
Jean (2001)	Review	Medullary swallowing CPG, DSG/VSG, NTS, NA	No	Swallowing‐mechanism evidence	Provides the classical framework for medullary swallowing control. Foundational but not PSD‐ or acupuncture‐specific.
Ertekin and Aydogdu (2003)	Review	Neurophysiology of swallowing	No	Swallowing‐mechanism evidence	Supports sensory integration, brainstem CPG organization, and cranial motor output in swallowing. Does not address acupuncture or neuroimmune mechanisms.
Prosiegel et al. (2005)	Clinical‐anatomical study	Localization of swallowing CPG and brainstem‐related dysphagia	Yes	Direct swallowing‐location evidence	Supports the importance of lesion location, especially brainstem involvement, in dysphagia phenotype. Does not address acupuncture or immune mechanisms.
Bolser et al. (2015)	Review	Dorsal medulla, NTS, cough‐swallow coordination, airway protection	No	Swallowing‐mechanism evidence	Supports the NTS and adjacent medullary regions as integrative nodes for airway protection. Not PSD‐ or acupuncture‐specific.
Pitts et al. (2018)	Animal experiments	Dorsomedial medulla, swallow pattern generation, respiratory–swallow interaction	Yes	Swallowing‐mechanism evidence	Supports respiratory–swallow phase coordination and phase‐sensitive medullary control. Animal evidence; not a poststroke acupuncture model.
Amorim et al. (2019)	Rat experiments	NTS neuroinflammation and reflex dysfunction	No	NTS/autonomic‐reflex neuroimmune evidence	Suggests that NTS inflammation can alter brainstem reflex function. Not swallowing‐specific and should not be interpreted as direct PSD evidence.
Mastitskaya et al. (2020)	Rat experiments	NTS astrocytes, vagal afferents, ATP/P2Y1 signaling	No	NTS/autonomic‐reflex glial evidence	Supports glial modulation within NTS‐related reflex circuits. Outcome focused on baroreflex rather than swallowing.
Duan et al. (2024)	Review	Brain‐peripheral immune crosstalk after stroke	No	Indirect stroke‐neuroimmune evidence	Supports the possibility that stroke‐related immune responses affect remote brain regions and recovery conditions. Does not directly assess swallowing CPG or acupuncture.
Cao et al. (2021)	Review	Acupuncture mechanisms in ischemic stroke	No	Acupuncture/EA stroke‐mechanism evidence	Summarizes acupuncture‐related effects on inflammation and neural repair after stroke. Indirect for PSD because swallowing is not the primary endpoint.
Kuang et al. (2024)	Review	Immunomodulatory mechanisms of acupuncture in ischemic stroke	No	Acupuncture/EA stroke‐neuroimmune evidence	Supports biological plausibility for acupuncture‐related immune modulation after stroke. Does not confirm NTS‐mediated swallowing recovery.
Fu et al. (2024)	Review	Acupuncture, endogenous neuroprotection, BBB/NVU‐related mechanisms	No	Acupuncture/EA stroke‐mechanism evidence	Supports possible effects of acupuncture on neurovascular and microenvironmental conditions after stroke. Does not directly assess swallowing outcomes.
Fang et al. (2023)	Review	Vagus nerve stimulation and neuroimmunomodulation	No	VNS‐related indirect evidence	Supports the modifiability of vagus‐related immune regulation and the vagal afferent‐NTS interface. VNS is related but not equivalent to acupuncture.
Long et al. (2022)	Cerebral ischemia model rats	taVNS, white matter repair, dysphagia symptoms	Yes	VNS‐related dysphagia evidence	Supports the translational relevance of vagus‐related stimulation in dysphagia recovery. Not an acupuncture study.
Yuan et al. (2022)	Rat experiments	CV23 acupuncture, PVH, PBN/NTS pathways, swallowing function	Yes	Preclinical acupuncture‐swallowing pathway evidence	Suggests acupuncture may influence swallowing through supramedullary‐brainstem pathways involving NTS‐associated circuits. Animal evidence with limited clinical translation.
Ye et al. (2024)	Rat PSD model	EA, M1, NTS, swallowing function	Yes	Direct preclinical PSD‐acupuncture evidence	Provides key preclinical support for NTS participation in EA‐related modulation of PSD. Does not fully establish neuroimmune or glial mediation.
Liu et al. (2024)	RCT protocol	Acupuncture, rs‐fMRI, DTI, PSD	Planned	Clinical PSD acupuncture protocol	Represents a move toward mechanism‐oriented clinical validation using neuroimaging and swallowing outcomes. Protocol only; no outcome data.
H. Guo et al. (2024)	Scoping review	Acupuncture evidence map, acupoint distribution, guideline support	Mixed	Clinical acupuncture evidence synthesis	Summarizes clinical evidence, acupoint patterns, and research gaps in acupuncture for PSD. Heterogeneity remains high and mechanistic depth is limited.
H. Li et al. (2024)	Systematic review and meta‐analysis	Acupuncture for aspiration caused by PSD	Yes	Clinical acupuncture evidence synthesis	Suggests potential benefit for PSD‐related aspiration. Included studies were of limited quality and mechanisms were not directly assessed.
Kang et al. (2025)	Randomized controlled trial	EA for poststroke oropharyngeal dysphagia	Yes	Clinical EA evidence	Suggests EA may provide additional benefit when combined with rehabilitation in PSD. Does not test NTS‐neuroimmune mechanisms.
F. Xu et al. (2025)	Randomized controlled trial	Acupuncture combined with conventional rehabilitation for PSD	Yes	Clinical acupuncture plus rehabilitation evidence	Supports acupuncture as a potential adjunct to conventional swallowing rehabilitation. Does not assess respiratory–swallow phase stability, NTS activity, immune markers, or BBB/NVU outcomes.
Yan et al. (2025)	Retrospective clinical study with propensity score matching	Vagus‐targeted stimulation, inflammatory markers, swallowing recovery	Yes	VNS‐related clinical dysphagia evidence	Supports the relevance of vagus‐related stimulation and inflammatory markers in dysphagia recovery. Not acupuncture; mechanistic chain remains incomplete.

Abbreviations: BBB, blood–brain barrier; CAP, cholinergic anti‐inflammatory pathway; CPG, central pattern generator; DSG, dorsal swallowing group; DTI, diffusion tensor imaging; EA, electroacupuncture; M1, primary motor cortex; NA, nucleus ambiguus; NTS, nucleus tractus solitarius; NVU, neurovascular unit; PBN, parabrachial nucleus; PSD, poststroke dysphagia; PVH, paraventricular hypothalamus; RCT, randomized controlled trial; rs‐fMRI, resting‐state functional magnetic resonance imaging; taVNS, transcutaneous auricular vagus nerve stimulation; VNS, vagus nerve stimulation; VSG, ventral swallowing group.

### 2.2 Evidence Classification and Interpretive Approach

Direct mechanistic evidence linking acupuncture, NTS activity, neuroimmune regulation, and swallowing recovery after stroke remains limited. To reduce overinterpretation, the evidence was interpreted according to its proximity to the proposed PSD framework. Studies that directly assessed swallowing outcomes after stroke were treated as direct PSD evidence. Studies examining swallowing CPG organization, NTS–NA circuitry, respiratory–swallow coordination, or brainstem reflex control were considered swallowing‐mechanism evidence, even when acupuncture was not involved. Studies addressing poststroke neuroinflammation, glial responses, BBB/NVU dysfunction, or autonomic dysregulation without swallowing‐specific outcomes were interpreted as indirect stroke‐mechanism evidence. Acupuncture and electroacupuncture studies in ischemic stroke models without dysphagia‐specific endpoints were used to support biological plausibility, not to establish a causal pathway for PSD recovery. Evidence from vagus nerve stimulation was considered mechanistically relevant but not equivalent to acupuncture, given the differences in stimulation sites and afferent recruitment patterns.

This interpretive evidence classification guided the use of the literature throughout the review. It was not a formal evidence‐grading procedure, and no recognized grading framework was applied. Direct evidence was used to support PSD‐related statements, whereas related but indirect mechanistic evidence was used only to formulate testable, evidence‐informed hypotheses regarding swallowing‐network vulnerability, neuroimmune regulation, and potential acupuncture‐related modulation. Connections for which no direct or indirect empirical support was identified were explicitly described as speculative possibilities rather than components of an established mechanistic pathway.

## 3 Brainstem Swallowing Network and Its Vulnerability to Poststroke Neuroimmune Disturbance

### 3.1 Stroke Location and Heterogeneity of Poststroke Dysphagia

PSD is not a single lesion‐defined condition. Its clinical phenotype depends strongly on stroke location, the rostrocaudal and mediolateral extent of the lesion, and the integrity of compensatory pathways. In lateral medullary infarction, dysphagia should not be attributed automatically to direct NTS injury. Lesions may variably involve or disconnect components of the dorsal swallowing group (DSG) and ventral swallowing group (VSG), the medullary reticular formation, NA‐related motor pathways, and the connections among these structures. Reported abnormalities include impaired swallowing‐pattern formation and event sequencing, asymmetric pharyngeal weakness or unilateral pharyngeal palsy, impaired upper esophageal sphincter opening or cricopharyngeal dysfunction, pharyngeal residue, and aspiration. The resulting phenotype and severity vary across patients according to lesion level and extent (Prosiegel et al. 2005;).

Supratentorial stroke can also cause dysphagia, but the mechanisms are usually less anatomically direct. Cortical and subcortical lesions may disturb volitional swallowing control, corticobulbar modulation, sensorimotor integration, attention, and respiratory–swallow coordination (González‐Fernández et al. 2013; Labeit et al. 2024). In these patients, medullary swallowing structures may remain structurally preserved. However, their output may still become less stable when descending regulation, sensory feedback, autonomic background state, or systemic inflammatory burden is altered.

The NTS‐centered framework proposed in this review should therefore be interpreted as complementary to lesion‐based models rather than as a replacement for them. In brainstem stroke, swallowing dysfunction may result from direct injury to, or disconnection among, components of the distributed medullary swallowing network; direct NTS involvement represents only one possible lesion pattern. In supratentorial stroke, instability within the medullary swallowing network is more likely to arise indirectly through disrupted descending control, altered sensory gain, respiratory–swallow phase mismatch, autonomic dysregulation, or neuroimmune stress. This distinction helps avoid overgeneralization and supports future studies that stratify PSD patients by lesion location and physiological phenotype.

### 3.2 Swallowing CPG Organization and Rhythmic Stability

Swallowing is not controlled by a simple “motor execution” process (Ertekin and Aydogdu 2003). Rather, it depends on a highly ordered, phase‐locked pattern of neural activity organized by the medullary CPG (Prosiegel et al. 2005). The medullary swallowing CPG comprises interconnected DSG and VSG. The DSG, located mainly within the NTS and adjacent dorsal medullary reticular formation, contributes to sensory integration and premotor pattern organization. The VSG, located in the ventrolateral medulla near the NA, coordinates the sequential activation of cranial motor nuclei and the oropharyngeal, laryngeal, and esophageal musculature, thereby generating the ordered sequence of swallow initiation, airway protection, bolus propulsion, and resumption of respiration (Ertekin and Aydogdu 2003; Jean 2001).

From a network perspective, the swallowing CPG has three organizational features that help explain its vulnerability. First, rhythm generation depends on a finely tuned balance between excitation and inhibition (E/I) (Pitts et al. 2018). Transitions among pharyngeal initiation, laryngeal closure, upper esophageal sphincter opening, and brief respiratory inhibition must occur within millisecond‐scale time windows (Pitts et al. 2018). Even subtle disturbances in membrane excitability, synaptic transmission, or inhibitory interneuron function may therefore lead to phase discoordination and drift in the motor sequence. Second, swallowing and respiratory rhythms are linked within the medulla through shared and cross‐coupled inhibitory circuits (Bolser et al. 2015). Swallowing is usually embedded in the expiratory phase and accompanied by respiratory suppression. This reflects not simply “behavioral coordination” but coordination between rhythmic neural networks at the phase level (Bolser et al. 2015). Once swallowing network stability declines, respiratory–swallow synchronization is often one of the first functions to deteriorate, reducing the reliability of airway protection and increasing aspiration risk. Third, the brainstem swallowing network has relatively limited functional redundancy. Although bilateral medullary hemi‐rhythm generators exist and achieve synchrony through crossed connections, the swallowing network lacks the extensive alternative pathways seen in cortical systems (D'Alatri et al. 2025). Its overall output depends on coordinated activity across the distributed dorsal and ventral medullary swallowing circuits. When local microenvironmental changes reduce the firing reliability of key nodes, rapid compensatory reconfiguration through collateral pathways is unlikely.

The swallowing network is therefore best viewed as a phase‐sensitive system. Ionic, glial, or BBB‐related disturbance may reduce output reliability. This helps explain why PSD can behave as a state‐dependent network disorder rather than a fixed motor deficit.

### 3.3 NTS and NA as Inflammation‐Sensitive Nodes Within the Medullary Swallowing Network

The NTS and nucleus NA should not be interpreted as a simple linear axis that fully accounts for the swallowing CPG. Swallowing is organized by a distributed medullary network that includes the DSG and VSG, reticular formation, sensory afferent pathways, and multiple cranial motor nuclei. Within this network, the NTS is mainly involved in sensory integration and premotor organization, whereas the NA contributes to motor output for pharyngeal and laryngeal muscles (Jean 2001; Ertekin and Aydogdu 2003; D'Alatri et al. 2025). In this review, the term “NTS‐centered” refers to the analytical focus of the proposed framework rather than to the NTS as the sole anatomical center of the swallowing CPG.

This distinction is important for PSD. In brainstem stroke, swallowing impairment may reflect direct injury to the NTS, NA, medullary reticular formation, or related afferent and efferent pathways. In supratentorial stroke, these medullary structures may remain anatomically preserved, but their function may still be influenced by altered descending control, sensory feedback, respiratory timing, autonomic state, or systemic inflammatory burden. Therefore, the NTS‐centered framework does not imply that all PSD phenotypes arise from a single lesion pattern or from a fixed NTS‐to‐NA pathway.

The relevance of the NTS lies in its convergent position. It receives glossopharyngeal and vagal afferent input related to swallowing, airway protection, visceral sensation, and autonomic regulation. This anatomical and functional convergence may make NTS‐level integration sensitive to changes in synaptic gain, extracellular ionic balance, cytokine exposure, and glial regulation. The NA, by contrast, represents one of the major motor‐output components for pharyngeal and laryngeal activity. Changes in premotor drive or local excitability may therefore affect the timing of airway protection, but such effects should be interpreted within the broader medullary swallowing network rather than as an isolated NTS–NA mechanism (Holt 2022; MacDonald and Ellacott 2020; Martinez and Kline 2021).

Altered glial and inflammatory regulation within or around the NTS is presented here as one possible, but unconfirmed, explanation for swallowing‐network vulnerability after stroke, rather than as a necessary or sufficient mechanism of PSD. Astrocytes and microglia in the dorsal vagal complex and adjacent brainstem regions participate in neurotransmitter clearance, ionic buffering, synaptic modulation, and inflammatory signaling. Evidence from NTS‐related autonomic and reflex studies suggests that inflammatory or glial changes can alter brainstem reflex function (Amorim et al. 2019; Mastitskaya et al. 2020; Troadec et al. 2022). However, these studies did not assess swallowing, and their relevance to swallowing CPG function is therefore indirect. Accordingly, the proposed NTS‐related glial link is treated as an evidence‐informed but indirect hypothesis rather than an established cause of PSD.

This interpretation places the NTS and NA within a distributed, lesion‐sensitive model of PSD. The NTS may act as a convergence node through which sensory, autonomic, respiratory, and immune‐related signals influence swallowing‐network stability. The NA and other motor nuclei contribute to the final patterned output. Future studies should test whether inflammatory or glial changes in these brainstem regions are associated with delayed swallow initiation, respiratory–swallow variability, or impaired airway protection.

### 3.4 Remote Neuroinflammation and Brainstem Swallowing Control

Even in supratentorial stroke, significant dysphagia may occur despite the absence of imaging evidence for direct structural damage to the NTS or NA (González‐Fernández et al. 2013). This mismatch between structural preservation and functional impairment suggests that swallowing dysfunction may reflect state‐dependent instability involving both interregional regulation and the local microenvironment (Duan et al. 2024).

Stroke can trigger both systemic and central immune responses and reshape the microenvironment of remote brain regions through several pathways, including elevated circulating inflammatory mediators, altered BBB permeability, immune cell trafficking, and brain–peripheral immune crosstalk (Duan et al. 2024). For a brainstem rhythmic network that depends on precise phase coordination, such disturbances do not need to produce visible structural damage to impair function (Bourhy et al. 2022). By altering membrane excitability, synaptic integration, and glial regulatory states, they may instead push the swallowing CPG into a low‐reserve, highly fluctuating mode, predisposing it to delayed onset, symptom fluctuation, or incomplete recovery (H. Wang et al. 2023).

In addition to remote neuroinflammation, stroke is often accompanied by altered central autonomic regulation, including reduced vagal tone and remodeling of the sympathetic–parasympathetic balance (Cha et al. 2023). Because the swallowing network is tightly coupled to respiratory rhythm and autonomic circuits, abnormal autonomic signaling may indirectly impair swallowing timing and stability by altering respiratory phase context, reflex thresholds, and network gain. This offers a more testable explanation for the clinical observation that dysphagia often coexists with increased risk of respiratory complications and autonomic instability (Pitts et al. 2018).

PSD may therefore be better viewed as a lesion‐dependent disorder with variable brainstem‐network vulnerability. Remote neuroinflammation and reduced autonomic reserve may further modify this vulnerability, especially when structural injury, impaired descending control, or respiratory–swallow discoordination is present (Duan et al. 2024). Thus, lesion location should be interpreted together with physiological and inflammatory markers.

## 4 Neuroimmune–Glial–Vascular Disturbances as Candidate Contributors to Swallowing‐Network Vulnerability

The following section considers neuroimmune, glial, and vascular disturbances as possible contributors to swallowing‐network vulnerability after stroke. To our knowledge, no study has directly demonstrated that poststroke neuroimmune, glial, or vascular alterations impair swallowing CPG function. The proposed relationships are therefore extrapolated from general ischemic stroke models, NTS‐related autonomic and reflex studies, and experimental research on neuroinflammation, glial regulation, and BBB/NVU dysfunction. These lines of evidence provide biological plausibility for the proposed framework but do not establish a causal pathway for PSD. Accordingly, the mechanisms discussed below should be interpreted as speculative, testable possibilities rather than confirmed determinants of swallowing dysfunction.

### 4.1 DAMPs‐TLR4/NF‐κB Signaling and Network Instability

Neuroinflammation after ischemic stroke is not simply an extension of a focal event. Rather, it may begin with local injury signals, be amplified through key receptor–transcriptional pathways, and provide a molecular starting point for subsequent vulnerability in remote brainstem swallowing networks (Shichita et al. 2012). After ischemia/reperfusion‐induced cellular injury and necrosis, the release of damage‐associated molecular patterns (DAMPs) increases. These include endogenous danger signals such as high mobility group box 1 (HMGB1), adenosine triphosphate (ATP), and heat shock proteins (HSPs). Once recognized by the innate immune system within the central nervous system, they initiate an inflammatory cascade (Gülke et al. 2018).

Among pattern‐recognition receptors, Toll‐like receptor 4 (TLR4) is widely regarded as a key hub in poststroke inflammatory amplification. TLR4 is abundantly expressed on microglia and astrocytes, where it recognizes multiple classes of DAMPs and triggers downstream signaling (Jin et al. 2013). Upon activation, TLR4 engages the nuclear factor kappa B (NF‐κB) transcriptional program through adaptor proteins such as myeloid differentiation factor 88 (MyD88). This promotes the expression of proinflammatory cytokines, including tumor necrosis factor‐α (TNF‐α) and interleukin‐1β (IL‐1β), and may sustain an inflammatory amplification loop (Shichita et al. 2012).

DAMPs–TLR4/NF‐κB signaling is not necessarily confined to the ischemic core. After stroke, inflammatory mediators may affect remote brain regions through humoral circulation, BBB dysfunction, and network propagation, thereby increasing immune signaling input and glial reactivity in regions not directly exposed to ischemia (Nishibori et al. 2020). For swallowing, the relevance of this pathway remains inferential. It may be most plausible in patients whose swallowing network is already vulnerable because of lesion location, impaired descending control, sensory disruption, or respiratory–swallow discoordination. In such cases, persistent inflammatory signaling could further reduce the reserve of phase‐sensitive brainstem circuits by altering excitability, synaptic integration, or glial regulation. NF‐κB signaling is also linked to glial state. Sustained activation may favor proinflammatory microglia and reactive astrocytes, weakening glutamate clearance, ionic buffering, and metabolic support (Jin et al. 2013). In PSD, TLR4/NF‐κB‐related activity should be interpreted as a stratification clue, not as proof of direct causality.

### 4.2 Microglial Activation, Threshold Drift, and Phase Instability

Poststroke microglial responses may influence more than local inflammatory signaling. By modifying cytokine release, receptor expression, and the synaptic microenvironment, reactive microglia could plausibly affect excitability thresholds within vulnerable neural circuits (Marciante et al. 2024; M. Wang et al. 2022). Following the release of DAMPs, microglia shift from a homeostatic surveillance state to a highly reactive state. This is accompanied by changes in secretory profile and receptor expression, contributing not only to inflammatory amplification but also to synaptic microenvironment remodeling and circuit gain regulation (S. Zhang 2019).

The swallowing CPG depends on coordinated excitation–inhibition balance and stable phase transitions. General ischemic stroke research indicates that microglial activation can alter cytokine signaling, receptor expression, synaptic transmission, and the local neural microenvironment (S. Zhang 2019; S. Xu et al. 2020). Separate NTS‐focused studies suggest that local neuroinflammation may modify brainstem reflex function, although the assessed outcomes were cardiovascular rather than swallowing‐related (Amorim et al. 2019). However, we did not identify studies that directly measured poststroke microglial activation within swallowing‐related NTS or medullary circuits together with changes in swallowing pattern generation or respiratory–swallow coordination. Accordingly, the proposed link between microglial reactivity and swallowing CPG instability remains speculative and should be regarded as one testable explanation rather than a demonstrated mechanism of PSD. Microglial responses after stroke are spatially and temporally heterogeneous (S. Zhang 2019). Separately, experimental work has shown that systemic inflammation can induce neuroinflammatory changes within the NTS and alter cardiovascular reflexes (Amorim et al. 2019). However, these studies did not examine poststroke swallowing or swallowing‐related medullary circuits. Whether poststroke inflammatory signals activate microglia specifically within swallowing‐related NTS or adjacent reticular regions, and whether such activation reduces swallowing‐network reserve, has not been demonstrated. This remains a testable possibility rather than an established explanation for fluctuating or delayed PSD.

Microglial activation is not uniformly harmful, and some responses may support repair once inflammatory burden declines (M. Wang et al. 2022). Whether persistent microglial activation is associated with greater respiratory–swallow variability or a differential response to neuromodulation remains to be tested.

### 4.3 Astrocytic Homeostasis and Swallowing Network Resilience

In contrast to the predominant role of microglia in initiating and amplifying inflammation, astrocytes are more likely to shape the resilience of rhythmic networks through homeostatic maintenance and metabolic support. In this way, they may determine whether the swallowing CPG can sustain stable output under inflammatory conditions. Astrocytes mediate glutamate clearance through high‐affinity transporters and contribute to extracellular potassium buffering and metabolic support. These functions are especially important in high‐frequency, phase‐dependent brainstem rhythmic networks (MacDonald and Ellacott 2020; Martinez and Kline 2021). When astrocytic homeostatic function is impaired, local accumulation of excitatory neurotransmitters and fluctuations in ionic homeostasis become more likely. This weakens the conditions required for phase locking and increases the risk of rhythmic drift.

Reactive astrogliosis is a common response to ischemic and inflammatory conditions. In the short term, astrocyte activation may be protective by buffering excess neurotransmitters, limiting inflammatory spread, and participating in tissue repair. However, when inflammatory signaling persists or is reinforced by the proinflammatory state of microglia, astrocytes may undergo a functional shift in homeostatic maintenance (S. Xu et al. 2020; Zhao et al. 2025). This may appear as reduced glutamate clearance, impaired ionic buffering, or abnormal coupling with the NVU, thereby translating inflammatory burden into reduced network resilience.

Astrocytes also secrete factors such as brain‐derived neurotrophic factor and nerve growth factor, which may support repair and plasticity. These effects depend on a reversible or homeostatic state (Y. Guo et al. 2025). Persistent maladaptive reactivity may limit recovery. Swallow‐initiation threshold and phase variability could help test this link.

### 4.4 BBB/NVU Instability and Peripheral–Central Immune Crosstalk

Persistent peripheral–central immune crosstalk after stroke is closely related to dysfunction of the BBB, which serves as a critical interface (Duan et al. 2024). Acute ischemia can increase BBB permeability through enhanced oxidative stress, endothelial dysfunction, and alterations in tight junction proteins (Nishibori et al. 2020). This facilitates entry of peripheral inflammatory signals into the central microenvironment and reinforces local glial responses.

BBB abnormalities are not necessarily confined to the ischemic core and peri‐infarct region (Nishibori et al. 2020). Stroke‐induced vascular inflammatory responses and neuroimmune signals may spread along the NVU and through neural networks to affect remote regions, including brainstem circuits involved in reflex and autonomic regulation (Bourhy et al. 2022). Whether this process directly affects swallowing CPG function remains unclear. A plausible interpretation is that BBB/NVU instability may increase humoral inflammatory input and vascular homeostatic stress in patients with already vulnerable swallowing circuits, thereby reducing tolerance to secondary physiological burdens.

Increased BBB permeability also facilitates migration of peripheral immune cells into the central nervous system (Duan et al. 2024). Once circulating monocytes, neutrophils, and lymphocytes enter the brain parenchyma, they can interact with local microglia and astrocytes, thereby altering inflammatory tone and the pace of tissue repair. Circulating cytokines and immune mediators may also influence central circuits through humoral pathways, leaving brainstem reflex networks chronically exposed to an unfavorable microenvironment (Duan et al. 2024).

Sustained peripheral–central crosstalk may constrain recovery by maintaining an activated immune state (Bourhy et al. 2022). Stable swallowing reorganization requires a permissive microenvironment (Bourhy et al. 2022). BBB/NVU‐related markers may therefore help identify patients with delayed recovery, symptom fluctuation, or less stable phase coordination.

Taken together, inflammatory amplification, microglial reactivity, astrocytic homeostatic changes, and BBB/NVU instability may create an unfavorable microenvironment for brainstem rhythm‐generating circuits after stroke. For PSD, this remains an inferential but testable proposition. The key question is not whether these mechanisms are unique to dysphagia, but whether they reduce the reserve of phase‐sensitive swallowing circuits in patients with lesion‐related or functional vulnerability. Future studies should examine whether neuroimmune–glial–vascular markers are associated with delayed swallow initiation, respiratory–swallow discoordination, residue, aspiration risk, or response to rehabilitation.

## 5 Acupuncture as a Potential Modulator of Poststroke Neuroimmune–Glial–Vascular Disturbances

### 5.1 Clinical Evidence for Acupuncture in Poststroke Dysphagia

Clinical studies and recent evidence syntheses suggest potential improvements in swallowing‐related outcomes in PSD, especially when combined with conventional swallowing rehabilitation. However, the certainty of the current evidence remains limited (Kang et al. 2025; H. Li et al. 2024; F. Xu et al. 2025).

Clinical protocols vary widely. Reported approaches include manual acupuncture, electroacupuncture, scalp acupuncture, tongue acupuncture, and peri‐laryngeal or submental acupoint stimulation. CV23/RN23 and other swallowing‐related points around the anterior neck, tongue, or submental region are frequently used, but acupoint selection remains heterogeneous (H. Guo et al. 2024). A recent randomized trial suggested that electroacupuncture at bilateral Jialianquan may provide additional benefit when combined with individualized rehabilitation in poststroke oropharyngeal dysphagia, although this evidence remains preliminary (Kang et al. 2025).

Outcome measures include bedside swallowing scales, water‐swallow tests, VFSS‐based indices, PAS, FOIS, swallowing‐related quality of life, nutritional indicators, and aspiration‐related complications. A recent systematic review reported potential benefits of acupuncture‐based interventions for aspiration related to PSD, but also emphasized low study quality and the need for more rigorous trials (H. Li et al. 2024). Interpretation of these findings is limited by several recurring methodological issues. Acupoint prescriptions, manual or electrical stimulation parameters, session frequency, and treatment duration vary substantially across studies. Control conditions range from usual rehabilitation to sham or non‐acupoint procedures, which limits comparability. Participant and practitioner blinding is inherently difficult in acupuncture trials, while assessor blinding and allocation procedures are not consistently reported. In addition, acupuncture is frequently combined with swallowing rehabilitation or other co‐interventions, making its independent contribution difficult to isolate. Outcome selection also varies from bedside scales and water‐swallow tests to instrumental measures such as VFSS‐based indices, PAS, and FOIS. These limitations reduce confidence in pooled estimates and restrict conclusions regarding the optimal protocol or underlying mechanism (H. Guo et al. 2024; H. Li et al. 2024). Thus, current clinical evidence supports acupuncture mainly as a potential adjunctive therapy. It does not yet confirm an NTS‐centered neuroimmune mechanism, because most studies have not measured respiratory–swallow coordination, NTS activity, autonomic tone, inflammatory markers, or BBB/NVU‐related outcomes alongside swallowing recovery.

### 5.2 Potential Effects of Acupuncture on Inflammatory Amplification

Poststroke neuroinflammation tends to amplify and persist, potentially reducing neural network reserve through cytokine signaling, glial reactivity, and alterations in BBB function (Kuang et al. 2024). In a range of ischemic stroke animal models, acupuncture or electroacupuncture has been reported to reduce NF‐κB‐related inflammatory activity (Cao et al. 2021). This is typically reflected by decreased NF‐κB nuclear translocation together with downregulation of proinflammatory mediators such as IL‐1β, TNF‐α, and interleukin‐6 (IL‐6).

Beyond downstream changes, some studies suggest that acupuncture or electroacupuncture may also influence DAMP signaling and Toll‐like receptor (TLR)‐mediated upstream amplification loops, thereby limiting early reinforcement and persistence of inflammatory positive feedback. These molecular changes are not confined to the peri‐infarct cortex. In some studies, they have also been observed in subcortical structures and brainstem‐related regions, suggesting that acupuncture may modulate interregional inflammatory input and glial reactive tone (Kuang et al. 2024).

At the cellular level, reductions in proinflammatory factors are often accompanied by attenuation of markers associated with reactive microglia and astrocytes (Liao et al. 2024). This suggests that acupuncture/electroacupuncture may indirectly influence neuron–glia interactions and network plasticity by reshaping the inflammatory microenvironment. At the clinical level, current evidence is largely correlational, linking changes in peripheral inflammatory markers with neurological outcomes (Zhu et al. 2025). Although this remains insufficient to establish a direct causal chain, it does support the idea that background conditions may be modifiable.

For PSD, the relevant question is whether inflammatory changes track with physiological proxies of swallowing‐network stability. Improvement in respiratory–swallow phase coupling or swallow‐initiation reliability would provide stronger mechanistic evidence than scale scores alone.

### 5.3 Potential Acupuncture‐Related Modulation of Glial State and Network Recovery

Poststroke responses of microglia and astrocytes may shape network recovery by altering the synaptic microenvironment, ionic homeostasis, and neurovascular interface function. In multiple ischemic stroke models, acupuncture/electroacupuncture has been reported to downregulate inflammatory mediators and immune activation signals in microglia, including TLR‐dependent pathways and NF‐κB‐mediated inflammatory cascades (Liao et al. 2024; Ren et al. 2024; Yang et al. 2025). These changes are accompanied by shifts in microglial morphology and transcriptional/protein expression profiles, collectively suggesting a move toward a less proinflammatory state (Liao et al. 2024).

Candidate upstream axes, including signal transducer and activator of transcription 6 (STAT6), peroxisome proliferator‐activated receptor γ (PPARγ), and annexin A1, may participate in inflammation resolution and repair (Liao et al. 2024; Ren et al. 2024). They are promising but remain indirect for PSD.

Compared with microglia, astrocytes are more likely to influence network resilience through homeostatic maintenance and metabolic support (Y. Guo et al. 2025; Pan et al. 2025). Current evidence suggests that acupuncture exerts coordinated effects on both microglial and astrocytic phenotypes, although the available evidence remains stronger for microglial modulation than for direct astrocyte‐targeted mechanisms (Y. Guo et al. 2025; Ren et al. 2024; Zhao et al. 2025). Microglia appear to be the primary targets for rebalancing immune state, whereas astrocytes help shape the microenvironment required for network recovery (Y. Guo et al. 2025; Yang et al. 2025).

The most useful readout is likely not a single glial marker. It is whether glial rebalancing is accompanied by less phase variability, more reliable swallow initiation, and better respiratory–swallow coordination (Lee et al. 2024). HRV and inflammatory markers may help stratify this response.

### 5.4 Potential Acupuncture‐Related Support of BBB/NVU Homeostasis and Microcirculation

Disruption of BBB and NVU homeostasis may allow sustained entry of peripheral inflammatory signals into the central nervous system and may reinforce glial reactivity, thereby maintaining an unfavorable microenvironment (Cao et al. 2021; Fu et al. 2024; Zhu et al. 2025). In animal models of ischemic stroke, acupuncture has been reported to attenuate BBB disruption and preserve NVU structural integrity (Chang et al. 2018; Dai et al. 2025; Fu et al. 2024). This is reflected by reduced cerebrovascular permeability, preservation of endothelial tight junction proteins, and downregulation of matrix metalloproteinase‐9 (MMP‐9) activity associated with BBB degradation (Dai et al. 2025; Fu et al. 2024).

Beyond these structural effects, acupuncture may also influence the function of multiple NVU components, including cerebral microvascular endothelial cells, perivascular astrocytic endfeet, and cerebral microcirculation (Fu et al. 2024; Zeng et al. 2024). Some preclinical studies suggest that acupuncture can improve local cerebral perfusion and enhance microcirculatory stability (Chang et al. 2018; Zeng et al. 2024). However, these effects are better interpreted as supportive stabilization of neurovascular function rather than complete reconstruction of fine neurovascular coupling.

By reducing vascular‐derived inflammatory input, BBB/NVU support may complement glial rebalancing (Cao et al. 2021; Zeng et al. 2024). These changes may promote a more permissive state for network recovery.

Current preclinical evidence points to three linked effects: weaker inflammatory amplification, less proinflammatory glial activity, and better BBB/NVU stability (Chang et al. 2018; Dai et al. 2025; Zhu et al. 2025). These effects should not be assigned to separate therapeutic pathways. They may act together by improving the brainstem microenvironment.

The main limitation is clear. Most evidence comes from ischemic stroke models in which swallowing was not the primary outcome (Zhu et al. 2025). For PSD, these studies support mechanistic inference, not a proven causal pathway.

## 6 NTS/Vagus‐Related Neuroimmune Interfaces and Recovery of the Swallowing Network

### 6.1 NTS as a Convergent Hub for Swallowing and Immune Regulation

The NTS is relevant to this review because it sits at the intersection of swallowing‐related sensory integration, autonomic regulation, and neuroimmune signaling. Afferents from the oral cavity, pharynx, larynx, and upper gastrointestinal tract converge in the NTS, mainly through glossopharyngeal and vagal pathways (D'Alatri et al. 2025; Ertekin and Aydogdu 2003; Holt 2022). The NTS also interacts with descending swallowing‐related inputs and contributes to both reflexive and volitional swallowing control (Ye et al. 2024). It is therefore better interpreted as an integrative gain node than as a passive sensory relay.

This position may help explain why supratentorial stroke can disturb pharyngeal initiation or respiratory–swallow coordination even when the medulla is structurally spared. In this setting, altered descending control, sensory feedback, autonomic tone, or inflammatory burden may reduce the stability of NTS‐related integration. Clinically, this may present as delayed swallow initiation, inconsistent airway protection, or respiratory–swallow discoordination.

The NTS should not, however, be regarded as a primary somatosensory target of acupuncture. Its dominant inputs are visceral and glossopharyngeal–vagal, rather than direct somatosensory projections. Acupuncture‐induced somatosensory input is more likely to influence NTS‐related swallowing networks indirectly, through multisynaptic brainstem, hypothalamic, parabrachial, cortical, or autonomic pathways. Experimental acupuncture studies in swallowing models suggest involvement of cortical swallowing‐related regions and NTS‐associated pathways, but do not establish a single direct somatosensory‐to‐NTS route (Yuan et al. 2022; Ye et al. 2024). This distinction helps define the boundary of the proposed acupuncture–NTS framework.

### 6.2 Vagal Afferents and the Cholinergic Anti‐Inflammatory Pathway

Vagal afferent signaling provides a major route by which visceral and immune‐related information reaches the NTS (Fang et al. 2023; Howland 2014; Ramos‐Martínez et al. 2021). The vagus nerve conveys mechanical, chemical, and inflammatory information from the pharynx, esophagus, gut, and other organs (J. H. Chen 2016). The NTS integrates these signals with descending inputs and coordinates downstream autonomic output.

The CAP offers a candidate link between vagal signaling and immune regulation (Fang et al. 2023; Lv et al. 2021; Pavlov and Tracey 2012). Vagus‐related pathways may suppress proinflammatory cytokine production through acetylcholine‐mediated mechanisms, including α7nAChR signaling (Ramos‐Martínez et al. 2021; Lv et al. 2021). Although peripheral immune organs contribute to the effector arm, the stability of the reflex depends partly on central integration within the NTS.

Vagus nerve stimulation and transcutaneous vagus nerve stimulation show that the vagal afferent—NTS–immune axis is modifiable (Fang et al. 2023; Howland 2014). Some studies report reduced neuroinflammation and improved outcomes after vagus‐related stimulation. Recent clinical work on dysphagia after stroke also suggests that vagus‐targeted stimulation may improve swallowing scores and reduce selected inflammatory markers (Yan et al. 2025). These findings support the translational relevance of the vagus–inflammation–swallowing axis, but they do not prove that acupuncture acts through the same route.

Acupuncture and vagus nerve stimulation should not be treated as equivalent. Their stimulation sites and recruitment patterns differ. Still, they may partially overlap at the level of somatic afferent input, NTS integration, autonomic balance, and immune regulation (Fang et al. 2023; Ramos‐Martínez et al. 2021). In PSD studies, inflammatory markers and heart rate variability (HRV) could be used as background variables. These variables may help test whether CAP‐related mechanisms shape response to acupuncture.

### 6.3 Proposed Model: Acupuncture, NTS Integration, and CPG Rhythmic Stability

The available evidence is limited in both number and scope. Directly relevant evidence is confined mainly to a small number of preclinical animal studies. In a rat model of PSD, Ye et al. (2024) reported that NTS neuronal activity and interactions between the NTS and the primary motor cortex participated in electroacupuncture‐related improvement of swallowing function. Yuan et al. (2022) provided related preclinical evidence that acupuncture at CV23 may influence swallowing through supramedullary–brainstem pathways involving the paraventricular hypothalamus, parabrachial nucleus, and NTS, although this was not a clinical PSD study. These studies support the participation of NTS‐associated neural circuits in acupuncture‐related swallowing modulation, but they did not determine whether neuroimmune or glial changes within the NTS mediated the observed effects. A separate body of ischemic stroke research has reported acupuncture‐ or electroacupuncture‐related changes in inflammatory signaling, glial responses, BBB/NVU integrity, and autonomic regulation; however, swallowing outcomes and NTS‐specific mediation were generally not assessed in these studies (Cao et al. 2021; Fu et al. 2024; Kuang et al. 2024; Y.‐W. Li et al. 2022). Vagus‐related stimulation studies provide additional indirect evidence that the vagal afferent–NTS–immune interface is modifiable, but these interventions are not equivalent to acupuncture (Fang et al. 2023; Long et al. 2022). No clinical study identified in this review directly demonstrated that NTS neuronal, neuroimmune, or glial modulation mediates acupuncture‐related swallowing recovery.

**FIGURE 1:**
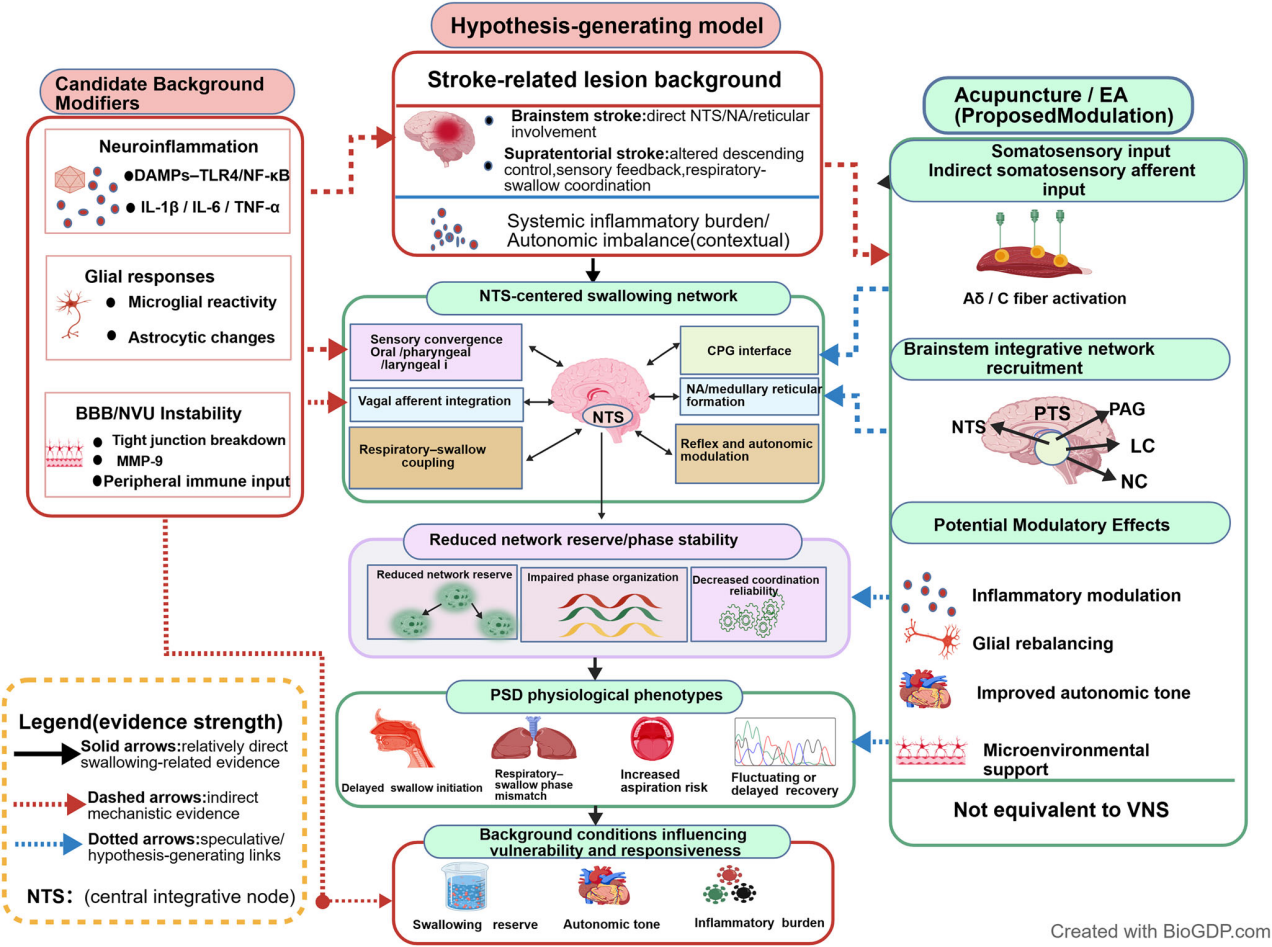
Hypothesis‐generating NTS‐centered framework linking poststroke dysphagia, neuroimmune regulation, and potential acupuncture‐related modulation. Poststroke dysphagia is presented as a lesion‐dependent condition with variable brainstem‐network vulnerability. Brainstem stroke may directly involve the NTS, NA, medullary reticular formation, and related pathways, whereas supratentorial stroke may influence swallowing indirectly through descending control, sensory feedback, respiratory–swallow coordination, autonomic regulation, and systemic inflammatory burden. Neuroinflammation, glial responses, BBB/NVU instability, and autonomic imbalance are shown as candidate background modifiers rather than established causes of swallowing CPG dysfunction. Acupuncture/electroacupuncture is proposed as a potential adjunctive intervention that may act through indirect somatosensory–brainstem pathways, autonomic regulation, inflammatory modulation, and microenvironmental support. In this figure, “NTS‐centered” denotes the analytical focus of the proposed framework and does not imply that the NTS is the sole anatomical or functional center of swallowing pattern generation; the medullary swallowing network comprises interacting dorsal and ventral swallowing circuits.

Based on this limited preclinical swallowing evidence and the broader indirect mechanistic literature, we propose a hypothesis‐generating model as summarized in Figure 1. Acupuncture‐induced somatosensory input may influence NTS‐related swallowing networks indirectly through multisynaptic brainstem, supramedullary, autonomic, or cortical pathways. At the same time, neuroimmune and autonomic modulation may improve the background conditions required for stable swallowing‐network output. If this model is valid, acupuncture‐related improvement should be accompanied by more reliable swallow initiation, less respiratory–swallow phase variability, and favorable changes in autonomic or inflammatory markers.

The model has clear boundaries. Acupuncture should not be equated with vagus nerve stimulation because their stimulation sites, afferent recruitment patterns, and central engagement differ. The proposed overlap is limited to downstream domains such as NTS integration, autonomic regulation, and immune signaling. Future studies should combine swallowing physiology, NTS‐centered neural measures, HRV, and inflammatory markers to test whether these domains change together and whether they mediate recovery in PSD.

## 7 Peripheral–Central Immune Interactions and Their Systemic Significance in PSD

### 7.1 Poststroke Brain–Peripheral Immune Imbalance and Its Impact on PSD

Stroke can reorganize brain–peripheral immune homeostasis. Acute inflammation may later shift toward stroke‐induced immunosuppression (Brea 2023; H. Wang et al. 2023). This trajectory is linked to infection risk, complications, and recovery (Brea 2023). It also provides a systemic background for dysphagia when brainstem structures appear preserved (Bourhy et al. 2022; Duan et al. 2024).

PSD may therefore evolve within a wider immune context. Elevated cytokines, altered leukocyte profiles, and higher systemic inflammatory burden are associated with poorer neurological outcomes and aspiration‐related complications (Brea 2023; H. Wang et al. 2023). These changes may amplify swallowing‐network vulnerability, especially during infection or metabolic stress (Bourhy et al. 2022; Duan et al. 2024).

Brainstem reflex nuclei are sensitive to peripheral immune signals (Pavlov and Tracey 2005). Cytokines and DAMPs may influence neuronal excitability through BBB‐related routes and vagal afferents (Duan et al. 2024; Pavlov and Tracey 2005). In the swallowing CPG, even modest immune stress may reduce phase organization and initiation reliability.

This view supports stratified research. Inflammatory burden and autonomic tone may help explain why patients with similar lesions recover differently (Brea 2023). They may also identify when neuromodulation is more likely to support swallowing rehabilitation.

### 7.2 Gut–Brain and Spleen–Brain Axes as Contextual Modulators

Peripheral immune organs may also shape the recovery background after stroke. Gut barrier disruption, microbiota‐immune interactions, and splenic immune mobilization can alter systemic immune tone (Benakis and Liesz 2022; Murthy et al. 2023; Wei et al. 2022). These pathways are best viewed as contextual modulators rather than direct drivers of dysphagia. They may influence whether the brainstem swallowing network operates in a permissive or high‐stress immune environment.

For PSD research, the practical value of these axes lies in stratification. Markers of gut barrier dysfunction, systemic inflammation, or splenic immune mobilization may help describe the broader immune burden (Benakis and Liesz 2022; Yu et al. 2021). Their relevance should be tested alongside HRV and phase‐stability outcomes, rather than assumed as a direct swallowing mechanism.

### 7.3 Potential Systemic Immune Modulation by Acupuncture and Its Relevance to PSD

In the context of persistent immune stress and increased susceptibility to secondary burdens after stroke, acupuncture is increasingly viewed as a neuromodulatory intervention that may influence systemic inflammatory tone and autonomic balance (Kuang et al. 2024; Ma et al. 2025). Experimental studies and clinical observations in ischemic stroke suggest that acupuncture/electroacupuncture may be associated with reductions in circulating proinflammatory mediators, alterations in immune cell composition, and improvements in autonomic tone indices (Kuang et al. 2024). These system‐level changes provide a biological basis for its potential contribution to functional recovery (Y. Zhang et al. 2024).

These systemic changes may matter for swallowing because peripheral immune signals can converge on the NTS through vagal pathways (Fang et al. 2023; Long et al. 2022; Yan et al. 2025). Lower inflammatory burden may reduce sustained input to the brainstem microenvironment. Improved vagal tone may also recruit endogenous anti‐inflammatory reflexes (Ma et al. 2025).

Still, systemic marker improvement is not proof of swallowing recovery. The stronger test is whether inflammatory and autonomic changes precede improved phase stability (F. Xu et al. 2025). This would support, but not prove, a pathway from systemic immune change to NTS integration and CPG stability.

Clinically, this pathway could help identify likely responders. Patients with high inflammatory burden and low vagal tone may require background rebalancing before training becomes effective. Patients with preserved phase stability may benefit mainly from synergy between acupuncture and behavioral swallowing therapy (F. Xu et al. 2025).

## 8 Translational Implications and Future Research Directions

### 8.1 Operationalizing Phase‐Stability Phenotypes in PSD

The proposed concept of phase stability needs to be linked to measurable swallowing phenotypes. The following measures should be interpreted as clinical or physiological proxies of network stability rather than as direct measures of swallowing CPG activity. Respiratory–swallow coordination can be assessed by identifying the respiratory phase before and after each swallow. The expiration–swallow–expiration pattern is generally considered more protective, whereas inspiration‐linked or highly variable patterns may indicate reduced airway‐protective coordination. Longer swallowing apnea and greater breath–swallow timing variability may provide additional markers of instability (Bolser et al. 2015; Pitts et al. 2018).

Swallow‐initiation reliability can be examined using VFSS or FEES. Relevant measures include delayed pharyngeal triggering, premature bolus spillage, vallecular or pyriform sinus residue before swallow onset, and variability across repeated swallows. In humans, CPG rhythmic stability cannot be measured directly in routine clinical settings. It may be approximated by temporal variability in hyoid movement, laryngeal vestibule closure, upper esophageal sphincter opening, swallowing apnea, and inter‐swallow intervals during repeated swallowing tasks (Ertekin and Aydogdu 2003; Labeit et al. 2024).

These physiological measures should be interpreted together with established clinical outcomes, including PAS, FOIS, VFSS‐ or FEES‐based residue scores, swallowing‐related quality of life, nutritional status, and aspiration pneumonia. In future acupuncture studies, their combination with respiratory monitoring, surface electromyography, HRV, and inflammatory markers may help determine whether clinical improvement is accompanied by more stable swallowing‐network output, rather than by changes in scale scores alone (Labeit et al. 2024; Aftyka et al. 2023).

### 8.2 Biomarkers for Stratification and Mechanistic Testing

PSD recovery is not determined only by lesion size or site. It may also depend on inflammatory burden, autonomic tone, and brainstem microenvironmental state (J. J. Zhang et al. 2022; Lago et al. 2025). Biomarkers are useful when they explain response heterogeneity and can be linked to swallowing physiology.

Circulating inflammatory markers are accessible but nonspecific. IL‐1β, IL‐6, and TNF‐α have been associated with poststroke outcomes and complications (Bacila et al. 2025). Serial trends may be more useful than single values because they capture persistent immune stress (Bacila et al. 2025).

Autonomic measures add a second layer. HRV is a noninvasive surrogate of vagus‐related regulation (Buitrago‐Ricaurte et al. 2020; Lago et al. 2025). When combined with respiratory–swallow phase indices, HRV may help connect systemic background to brainstem rhythm control.

Glial and neurotrophic markers are harder to measure directly in the brainstem (G. Chen et al. 2023; J. J. Zhang et al. 2022). A pragmatic design would combine fluid biomarkers with imaging, electrophysiology, and quantitative swallowing measures. The key test is temporal alignment between biomarker change and improved phase stability (Z. Wang 2025).

### 8.3 From Correlation to Causal Testing

Future work should test a sequence, not a single association: background conditions, NTS integration, and CPG phase stability. Studies should ask whether acupuncture improves swallowing by changing this sequence, or through other routes (Ye et al. 2024).

At the hub level, experiments should test whether NTS activity determines swallow initiation reliability and respiratory–swallow coupling (Broussard and Altschuler 2000; Liu et al. 2024). Targeted manipulation of NTS neuronal activity, combined with quantitative swallowing physiology, would help separate nodal causality from broader network correlation.

At the pathway level, vagal afferent‐NTS signaling deserves priority (Broussard and Altschuler 2000; Tsujimura et al. 2013; Ueda et al. 2016). Blocking, inhibiting, or activating this route could clarify whether acupuncture effects depend on intact peripheral afferent‐brainstem integration. Such work would also help distinguish overlap from difference between acupuncture and vagus nerve stimulation.

At the mediating level, microglial and astrocytic states within swallowing‐related brainstem nuclei should be tested when feasible. If glial manipulation changes CPG stability and acupuncture response, the model gains support. If not, it should be revised.

### 8.4 Toward Stratified Acupuncture Strategies

If PSD reflects a vulnerable brainstem network, acupuncture should be studied as a mechanism‐oriented adjunct. The key questions are for whom it works, under what background conditions, and with which physiological changes (G. Chen et al. 2023; Lago et al. 2025).

A practical design is a phenotype‐by‐background framework. Trials should measure respiratory–swallow phase coordination, swallow‐initiation reliability, transition stability, and aspiration‐related indices. Inflammatory markers and HRV can define the immune‐autonomic background.

This framework also changes how parameters are interpreted. Acupoint combinations, intensity, frequency, and treatment duration may recruit different afferent pathways and brainstem responses (H. Guo et al. 2024). Parameter optimization should therefore occur with background stratification and physiological outcomes, not in isolation (Liu et al. 2024).

Clinically, acupuncture should not replace behavioral swallowing rehabilitation. Swallowing training provides task‐specific input. Acupuncture may improve the conditions that make training more effective (H. Guo et al. 2024; F. Xu et al. 2025). This combined view fits neurorehabilitation practice and avoids overstatement.

## 9 Conclusion

PSD should not be viewed only as a downstream motor deficit. It may also reflect variable vulnerability of the distributed medullary swallowing network, within which the NTS serves as an important sensory‐integrative and premotor node. The stability of this network depends on coordinated swallowing pattern generation, respiratory coupling, descending control, and autonomic background state. Poststroke inflammation, altered glial responses, and BBB/NVU dysfunction may modify this vulnerability, although direct swallowing‐specific evidence remains limited.

Acupuncture may support recovery by modulating this vulnerable background. Its plausible effects include changes in inflammatory burden, glial state, autonomic balance, and microenvironmental permissiveness. These claims remain partly inferential because most mechanistic studies did not use swallowing as the primary outcome.

Future studies should test the pathway from background conditions to NTS integration and rhythmic stability. Stratifying patients by inflammatory and autonomic state, and using phase‐stability phenotypes as core outcomes, may make acupuncture research more useful for neurorehabilitation.

## Author Contributions

**Wei Li**: conceptualization, methodology, writing – original draft, writing – review and editing. **Lebin Liu**: conceptualization, supervision, writing – review and editing. **Yingying Tu**: literature curation, writing – review and editing. **Chongjiu Gong**: literature curation, writing – review and editing. **Xiao Ran**: visualization, writing – review and editing. **Jiuguo Pei**: supervision, project administration, writing – review and editing. All authors read and approved the final manuscript.

## Funding

The authors have nothing to report.

## Conflicts of Interest

The authors declare no conflicts of interest.

## Ethics Statement

The authors have nothing to report.

## Data Availability

No new data were generated or analyzed in this study. Data sharing is not applicable to this article.
